# Repetition of Computer Security Warnings Results in Differential Repetition Suppression Effects as Revealed With Functional MRI

**DOI:** 10.3389/fpsyg.2020.528079

**Published:** 2020-12-07

**Authors:** C. Brock Kirwan, Daniel K. Bjornn, Bonnie Brinton Anderson, Anthony Vance, David Eargle, Jeffrey L. Jenkins

**Affiliations:** ^1^Neuroscience Center, Brigham Young University, Provo, UT, United States; ^2^Department of Psychology, Brigham Young University, Provo, UT, United States; ^3^Department of Information Systems, Brigham Young University, Provo, UT, United States; ^4^Department of Management Information Systems, Fox School of Business, Temple University, Philadelphia, PA, United States; ^5^Leeds School of Business, University of Colorado Boulder, Boulder, CO, United States

**Keywords:** repetition suppression, fMRI, habituation, anterior insula, cybersecurity

## Abstract

Computer users are often the last line of defense in computer security. However, with repeated exposures to system messages and computer security warnings, neural and behavioral responses show evidence of habituation. Habituation has been demonstrated at a neural level as repetition suppression where responses are attenuated with subsequent repetitions. In the brain, repetition suppression to visual stimuli has been demonstrated in multiple cortical areas, including the occipital lobe and medial temporal lobe. Prior research into the repetition suppression effect has generally focused on a single repetition and has not examined the pattern of signal suppression with repeated exposures. We used complex, everyday stimuli, in the form of images of computer programs or security warning messages, to examine the repetition suppression effect across repeated exposures. The use of computer warnings as stimuli also allowed us to examine the activation of learned fearful stimuli. We observed widespread linear decreases in activation with repeated exposures, suggesting that repetition suppression continues after the first repetition. Further, we found greater activation for warning messages compared to neutral images in the anterior insula, pre-supplemental motor area, and inferior frontal gyrus, suggesting differential processing of security warning messages. However, the repetition suppression effect was similar in these regions for both warning messages and neutral images. Additionally, we observed an increase of activation in the default mode network with repeated exposures, suggestive of increased mind wandering with continuing habituation.

## Introduction

One major obstacle to computer security is habituation on the part of computer users to repeated computer security messages. Sometimes termed “warning fatigue,” this habituation to security warnings can result in lower rates of security behavior (Akhawe and Felt, 2013). At a biological level, repeated exposure to a stimulus results in repetition suppression, or a decreased neuronal response to that stimulus. Evidence for repetition suppression has been observed for both auditory (Costa-Faidella et al., 2011; Todorovic et al., 2011) and visual processing (Summerfield et al., 2008, 2011; Larsson and Smith, 2012) using recording methods including single-unit recording electrophysiology (Malmierca et al., 2009), functional magnetic resonance imaging (fMRI) (Larsson and Smith, 2012; Grotheer and Gyula, 2015), electroencephalography (Costa-Faidella et al., 2011; Summerfield et al., 2011), and magnetoencephalography (Todorovic et al., 2011; Todorovic and de Lange, 2012).

The effect of habituation has been studied in different ways in different fields. For example, in marketing, a great deal of research has studied “repetition effects” (Schmidt and Eisend, 2015), or the “differential effects of each successive advertising exposure, i.e., the differential effects of a given exposure within a sequence of exposures” (Pechmann and Stewart, 1988, p. 287). The most accepted theory explaining repetition effects is Berlyne’s (1970) two-factor theory that explains a “wear-in” process in which familiarity and ad effectiveness increases with repetitions, and a later “wear-out” process, in which the effectiveness of an advertisement decreases with each succeeding exposure.

In contrast, in the fields of warning science and computer security, repeated exposure to a warning does not lead to beneficial familiarity effects, but leads directly to diminished attention to a warning (Wogalter and Vigilante, 2006; Vance et al., 2017). In computer security, habituation to warnings has been frequently inferred as a factor without measuring it directly (Bravo-Lillo et al., 2014). For example, Akhawe and Felt (2013, p. 268) reported that the most common web browser SSL error had the lowest adherence rate, which they concluded was “indicative of warning fatigue.” However, some studies have examined habituation directly by measuring decreased attention to warnings using eye-tracking, mouse cursor tracking, and fMRI (Anderson et al., 2016a,b; Vance et al., 2018). The results from all of these studies show decrease attention to warnings after only 2–3 exposure. However, none of these studies directly compared how people habituate to computer security warning stimuli compared to general software application stimuli, a gap that this article investigates.

The underlying process of repetition suppression is not fully known and there is some debate as to the mechanisms that achieve the decrease in neuronal activation. One view is the bottom-up, or fatigue model, which suggests that differences in activity are related to the refractory period of local neural generators in response to physical stimulation (see Grill-Spector et al., 2006, for review). Another view is the top-down, or predictive coding, model which posits that repetition suppression is due to the expected probability of a stimulus recurring (Mayrhauser et al., 2014). Recent research gives support for the predictive coding model; Summerfield et al. (2008) found that the repetition suppression effect was modulated by an expectation of how often stimuli would repeat. Larsson and Smith (2012) also found that expectation can influence the repetition suppression effect, but only when one is actively attending to the repeated stimulus. Valentini (2011), however, observes that there is evidence for some contribution by both bottom-up and top-down processes in repetition suppression.

The response to repeated stimulus exposure is not uniform across the brain and may depend on context or task demands. Multiple areas in the occipital and temporal lobes demonstrate a repetition suppression effect (Kovacs et al., 2013; Mayrhauser et al., 2014). Structures in the medial temporal lobe (MTL) including the hippocampus also demonstrate decreased fMRI activation in response to repeated stimuli, sometimes referred to as a novelty response (Stern et al., 1996). On the other hand, other regions of the MTL demonstrate an increase in fMRI activation in response to repeated stimuli (Kirwan et al., 2009), referred to as a familiarity response (e.g., Daselaar et al., 2006). In a review of the repetition enhancement effect (increased fMRI activation with stimulus repetition), Segaert et al. (2013) identified several factors that influence whether repetition suppression or repetition enhancement is observed. These factors include task demands and cognitive processes engaged (including memory, learning, and attention). Further, regions in the default mode network (DMN), including the medial parietal lobe, inferior parietal lobule, and prefrontal cortex, also demonstrate an increase in fMRI activation with repeated stimulus exposure (Danckert et al., 2007; McDonald et al., 2010). This increase in DMN activation has been linked to inattention to a specific stimulus (Mason et al., 2007; Raichle and Snyder, 2007) as demonstrated by decreased subsequent recognition memory accuracy (Shrager et al., 2008). Based on these findings, it is reasonable to assume that repeated exposure to a stimulus will result in decreased activation in sensory and attention networks and increased activation in the DMN.

Studies of repetition suppression typically use only a few repetitions over a short period of time typically lasting only a few minutes (Chouinard et al., 2008; Summerfield et al., 2008, 2011). Further, while some studies of novelty and familiarity effects have demonstrated both effects in different regions of the MTL within the same paradigm (notably in the hippocampus; e.g., Daselaar et al., 2006), none have examined the longer-term trade-off between novelty and familiarity signaling in the same region within the same paradigm. Thus, it is unclear if these repetition suppression effects (i.e., decreases in fMRI activation) would continue with repeated exposures to the same stimulus in the same scanning session.

Another limitation of the current repetition suppression effect literature is that generally simple stimuli have been studied, such as tones (Costa-Faidella et al., 2011; Todorovic et al., 2011; Todorovic and de Lange, 2012) or single objects (Chouinard et al., 2008; Kovacs et al., 2013). More complex visual stimuli, such as faces, have been used as well (Summerfield et al., 2008, 2011; Larsson and Smith, 2012). However, it is not known how repetition applies to complex, everyday stimuli such as images of computer programs over repeated exposures, much like what is experienced during everyday computer use. Accordingly, images of common computer scenes provide a real-world application for the phenomenon of repetition suppression. Further, computer security warning messages have a learned, negative emotional content. Thus, the use of computer warning messages provides the opportunity to examine the effect of learned emotional stimuli in a more realistic setting.

Computer security warnings are not inherently aversive stimuli and thus any negative emotional valence associated with them must be learned, likely through social or verbal means. While much is known about the neural circuitry involved in classical fear conditioning, relatively little is known about the neural circuitry of social fear learning (Olsson and Phelps, 2007). Classical fear conditioning is critically dependent on the amygdala (Phillips and LeDoux, 1992) and has been shown to activate amygdala in human neuroimaging paradigms (Buchel and Dolan, 2000). Similarly, social and verbal fear learning have been shown to activate the amygdala (e.g., Phelps et al., 2001), indicating a general role of the amygdala in fear acquisition and fear expression in both classically conditioned and socially or verbally acquired fear responses. The anterior insula is also activated for verbally acquired fear representations (Phelps et al., 2001; Olsson and Phelps, 2007). Anterior insula activity has been linked to the anticipation of negative events (Grupe and Nitschke, 2013) and its dysfunction has been linked to avoidance of threat uncertainty (Paulus and Stein, 2006). Anterior insula activation has also been associated with general arousal levels, regardless of positive or negative valence of the stimulus (Knutson et al., 2014).

In the current experiment, we sought to examine the repetition suppression effect over repeated exposures to complex, everyday stimuli both generally and for socially constructed fearful stimuli. We anticipated a repetition suppression effect (i.e., decreased BOLD signal) in visual processing stream but increased activation in DMN regions with repeated exposures. We further examined the effect of repeated exposures on novelty and familiarity signals in the MTL. Finally, we investigated the effects of repetition on responses of brain regions associated with fear and/or arousal.

## Materials and Methods

### Subjects

Twenty-two participants (4 female, 18 male; 24 years old, range 20–27) were recruited from the university community and gave written informed consent prior to participation. The sample size was determined by previous literature in this area (Dimoka, 2012) and guidelines set forth by Desmond and Glover (2002) to calculate the required number of subjects to ensure adequate statistical power. Participants were right-handed native English speakers with normal or corrected-normal visual acuity. Participants self-reported free of psychiatric or neurological conditions. As members of the university community, these subjects had a high level of computer literacy. The experiment was approved by the University Institutional Review Board and was conducted in accordance with the principles of the Declaration of Helsinki. Participants were compensated US $25 for a 60 min session.

### Behavioral Task

**FIGURE 1:**
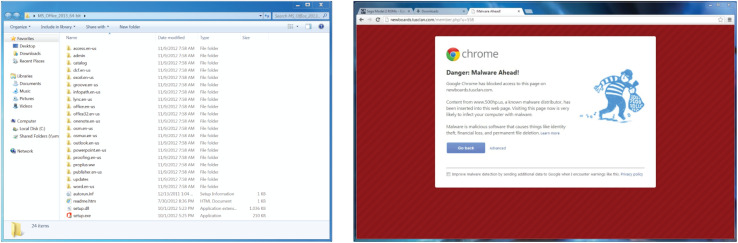
Example stimuli from the behavioral experiment. Participants viewed repetitions of general software images (left) and computer security warnings (right) in a randomized order.

We used an event-related, within-subject experimental design in which participants viewed a random sequence of 60 images of general software application screenshots (such as Microsoft Word, Excel, and other common applications) and security warnings collected by the researchers (Figure 1). The experiment utilized a variety of actual security warnings from programs running on a Windows operating system. Table 1 summarizes each type of warning.

**TABLE 1:** Description of warnings shown to participants.

**Warning type description**
The operating system warning that a program can “make changes to this computer”
A virus protection program warning “intruder detected”
A firewall warning “Danger: Malware Ahead!”
A firewall warning “blocked activity of harmful software”
Facebook warning of a potentially abusive link
A firewall warning that it has block some feature of a program
A web browser warning that a page contains non-secure items
A spreadsheet warning that a file contains macros
A web browser warning of a “Reported Web Forgery”
A program warning that an online application is attempting to access files on your computer
The operating system warning that an application is trying to run
The operating system warning that it cannot verify the publisher of a driver software
A browser warning that a connection is untrusted (SSL warning)
A virus protection program warning that a trojan was found

**FIGURE 2:**
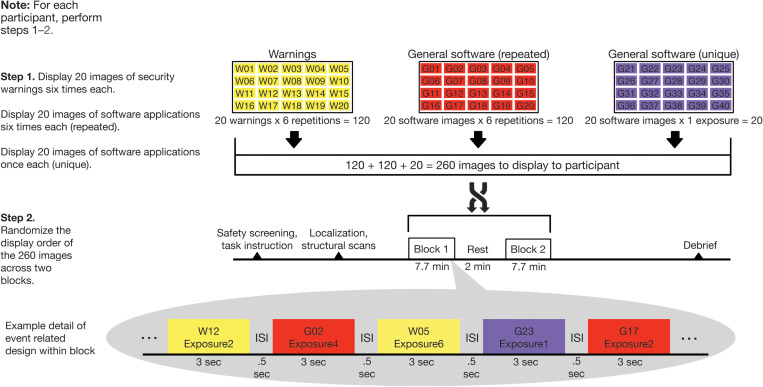
Randomization and behavioral task scheme. Computer security warning and general software images were displayed in two blocks of 130 images in random order. Twenty security warnings and 20 general software images were displayed six times each while 20 additional general software images were displayed once only.

For visual consistency, all images of general software applications and security warnings were for the Windows operating system. Our experimental design is graphically depicted in Figure 2 and consisted of two steps for each participant. In Step 1, images were organized into three sets of 20 images each. The first two sets comprised security warnings and general software applications. These were repeated six times each in random order across the duration of the scan. A third set consisted of general software application images, which were each displayed only once during the scan. This was done to create a baseline of unique presentations throughout the task. Thus, there were 260 total images (20 warnings × 6 repetitions + 20 software images × 6 repetitions + 20 software images × 1 exposure each) displayed in the experiment. In Step 2, the 260 images were randomized for each participant across two blocks of 7.7 min each (with a ∼2 min break in between).

Subjects were given a verbal briefing about the MRI procedures and the task, and then situated supine in the MRI scanner. Visual stimuli were displayed using E-prime software (version 2.0.10) and were viewed by means of a mirror attached to the head coil reflecting a large monitor outside the scanner. On each trial, images were displayed for 3 s each, with a 0.5 s inter-stimulus interval (ISI).

In order to keep participants attentive during the viewing of images, they were instructed to use an MR-compatible keypad to indicate if the image shown was common or uncommon in their experience. We intentionally used a simple task in order to minimize influence on the repetition suppression effect, while still enabling measurement of participant attention to the task. Such an approach is common in pattern separation tasks, for example, where the repetition suppression effect is used to differentiate repeated images from similar lures (Lacy et al., 2011). Participants responded on 96% of trials (*SD* = 10%), indicating that they were appropriately engaged and on task. At the end of the experimental task, participants were debriefed, compensated, and dismissed.

### Equipment and Scan Parameters

MRI scanning took place on a Siemens 3T Trio scanner. For each scanned subject, we collected a high-resolution structural MRI scan for functional localization in addition to the two functional scans. Structural images were acquired with a T1-weighted magnetization-prepared rapid acquisition with gradient echo (MP-RAGE) sequence with the following parameters: TE = 2.26 ms, flip angle = 9°, slices = 176, slice thickness = 1.0 mm, matrix size = 256 × 215, voxel size = 1 mm × 0.98 mm × 0.98 mm. Functional scans were acquired with a gradient-echo, echo-planar, T2^∗^-weighted pulse sequence with the following parameters: TR = 2,000 ms, TE = 28 ms, flip angle = 90°, slices = 40, slice thickness = 4.0 mm (no skip), matrix size = 64 × 64, voxel size = 3.44 mm × 3.44 mm × 3 mm. All data are available at https://openneuro.org/datasets/ds002363 and data analysis scripts are available at https://github.com/Kirwanlab/RepetitionSuppression.

### Analysis

MRI data were analyzed with the Analysis of Functional Images (AFNI) suite of programs (Cox, 1996). Briefly, structural and functional scans were converted to NIfTI file format using dcm2niix^1^ (Li et al., 2016) which performs slice time correction of functional scans as part of the conversion process. Motion correction of the functional runs was calculated based on the volume with the least amount of noise for each functional run. Spatial normalization was calculated for each T1-weighted structural scan to MNI space. The motion correction and spatial normalization transformations were concatenated so that functional data underwent a single interpolation, thus reducing blurring of the data in preprocessing (Muncy et al., 2017). Functional data were scaled by the mean signal intensity. An intersection mask was calculated based on the overlap of the extent of coverage of the T2^∗^-weighted functional scans and a gray matter mask of the MNI template brain. All group analyses were performed within this intersection mask.

For the first-level regression analysis, behavioral vectors were created that coded for stimulus type (e.g., security warnings, general software application screenshots) and repetition number. Additionally, we included a regressor for the single-presentation general computing screenshots to serve as a stimulus check to ensure that any observed decreases in responding were not due to fatigue. Stimulus events were modeled using a 3 s boxcar function convolved with the canonical hemodynamic response. Regressors coding for motion (6 regressors per scan run) and polynomial regressors coding for scan run and scanner drift were also entered into the model as nuisance variables. To control for size differences between the general software application screenshots and security warnings, the total size of each stimulus (in pixels) was also entered as a nuisance variable. Resulting beta values were blurred with a 5 mm FWHM Gaussian kernel. Beta values for the conditions of interest were then entered into the group-level analysis, which consisted of a model with stimulus type (two levels) and repetition number (six levels) as within-subject factors. The residuals from the first-level regression analysis were also blurred with a 5 mm FWHM Gaussian kernel and used to estimate the smoothness of each functional scan. This smoothness estimate was then entered into Monte Carlo simulations to determine a spatial extent threshold for performing corrections for multiple comparisons in group-level analyses (Cox et al., 2017). All tests were corrected for multiple comparisons using a voxel-wise threshold of *p* < 0.001 and a spatial extent threshold of 12 voxels, nearest-neighbors level 2 (overall *p* < 0.01).

## Results

**FIGURE 3:**
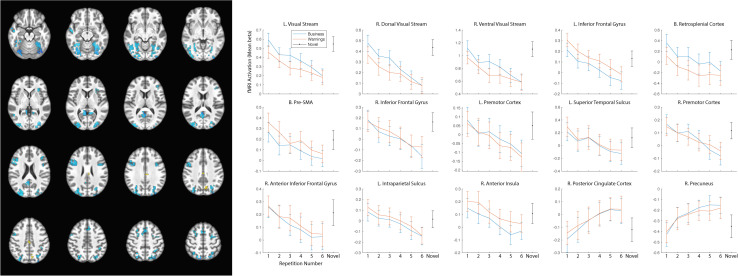
Significant clusters of activation demonstrating a main effect of repetition number displayed on axial slices (left). Regions with negative linear trends are indicated in cooler colors (blue) while regions with positive linear trends are indicated in warmer colors (yellow/orange). Mean betas for the general software and security warning conditions over six repetitions within each cluster are displayed on the right. Posterior clusters in the visual processing streams, and retrosplenial cortex displayed a main effect of stimulus type with greater activation for the general software images than security warnings. More anterior regions including the left inferior frontal gyrus, the pre-SMA, and the right anterior insula displayed a main effect of stimulus type with greater activation for security warnings than general software images. The linear trend was significant in all clusters, however only the PCC and precuneus displayed a positive trend. Activation for the single-presentation general computing screenshots was significantly different from the final presentation of the repeated images in each cluster for both conditions with the exception of the Warning stimuli in the right anterior insula. Error bars, SEM.

As our hypotheses concerned differential responses over repeated exposures to stimuli, we first identified clusters that showed a main effect of repetition. Fifteen clusters survived correction for multiple comparison: left and right dorsal and ventral visual processing streams, left and right inferior frontal gyrus (with a separate cluster in right anterior inferior frontal gyrus), bilateral presupplementary motor area (pre-SMA), bilateral retrosplenial cortex, left and right premotor cortex, left superior temporal sulcus, left intraparietal sulcus (IPS), right anterior insula, right posterior cingulate cortex (PCC), and right precuneus (see Table 2 for MNI coordinates and statistics and Figure 3 for locations and responses). Average betas for each of the stimulus type and repetition conditions were extracted from these clusters and subjected to follow-up analyses (repeated-measures ANOVAs and linear contrasts). The follow-up analysis revealed a significant linear trend of repetition (collapsing over stimulus type) in each of the clusters (*p*’s < 0.01), consistent with our hypotheses of sustained effects across numerous repetitions. All linear trends were negative except for the PCC and precuneus (see Figure 3, right panel). There was a main effect of stimulus type with greater activation for general software screenshots than security warnings (i.e., Business > Warning) in the left and right visual stream (dorsal and ventral), and the retrosplenial cortex (Table 2). The opposite effect (i.e., Warning > Business) was observed in clusters in the left inferior frontal gyrus, the pre-SMA, and the right anterior insula. The stimulus type by repetition number interaction was not significant in any cluster. Finally, there was a stimulus type by repetition number interaction in the linear trends in the left and right (dorsal) visual processing streams (Table 2).

**TABLE 2:** Location and description of significant clusters showing a main effect of repetition.

			**MNI coordinates**			**ME stimulus type**			**ME repetition**			**INTX stim. type × repetition**			**Linear trend INTX**		
							
**Label**	**#Voxels**	**Direction**	***X***	***Y***	***Z***	***F*(1,21)**	***p***	**η^2^_*p*_**	***F*(5,105)**	***p***	**η^2^_*p*_**	***F*(5,105)**	***p***	**η^2^_*p*_**	***F*(1,21)**	***p***	**η^2^_*p*_**
L. Visual	483	Negative	−46	−59	−8	24.699	**<0.001**	0.54	26.391	**<0.001**	0.557	2.113	0.07	0.091	4.791	**0.04**	0.186
R. Dorsal visual	213	Negative	29	−66	34	10.953	**0.003**	0.343	26.499	**<0.001**	0.558	2.23	0.057	0.096	5.566	**0.028**	0.21
R. Ventral visual	173	Negative	29	−80	−11	7.469	**0.012**	0.262	24.083	**<0.001**	0.534	1.992	0.086	0.087	2.745	0.112	0.116
L. Inferior frontal gyrus	124	Negative	−40	10	27	30.564	**<0.001**	0.593	24.964	**<0.001**	0.543	0.394	0.852	0.018	0.337	0.567	0.016
B. Retrosplenial cortex	90	Negative	−5	−52	16	87.007	**<0.001**	0.806	13.861	**<0.001**	0.398	1.615	0.162	0.071	3.069	0.094	0.128
B. Pre-SMA	58	Negative	−9	16	64	21.41	**<0.001**	0.505	13.054	**<0.001**	0.383	1.496	0.198	0.066	1.274	0.272	0.057
R. Inferior frontal gyrus	32	Negative	40	6	27	3.03	0.096	0.126	15.056	**<0.001**	0.418	1.396	0.232	0.062	1.151	0.296	0.052
L. Premotor cortex	32	Negative	−29	16	54	2.935	0.101	0.123	21.341	**<0.001**	0.504	0.407	0.843	0.019	0.228	0.638	0.011
L. Superior temporal sulcus	21	Negative	−53	−4	−15	1.338	0.26	0.06	21.313	**<0.001**	0.504	0.453	0.81	0.021	0.128	0.724	0.006
R. Premotor cortex	20	Negative	33	−4	58	0.019	0.891	0.001	11.598	**<0.001**	0.367	1.264	0.286	0.059	2.622	0.121	0.116
R. Anterior inferior frontal gyrus	19	Negative	40	30	23	1.792	0.195	0.079	11.098	**<0.001**	0.346	0.29	0.917	0.014	0.154	0.698	0.007
L. Intraparietal sulcus	15	Negative	−29	−59	54	3.143	0.091	0.13	14.602	**<0.001**	0.41	0.141	0.982	0.007	0.505	0.485	0.023
R. Anterior insula	14	Negative	29	27	3	17.297	**<0.001**	0.464	15.996	**<0.001**	0.444	0.571	0.722	0.028	0.05	0.826	0.002
R. Posterior cingulate cortex	14	Positive	5	−25	30	0.997	0.329	0.045	10.875	**<0.001**	0.341	0.2	0.962	0.009	0.469	0.501	0.022
R. Precuneus	13	Positive	9	−70	37	1.076	0.311	0.049	0.9114	**<0.001**	0.303	0.65	0.662	0.03	1.589	0.221	0.07

*Significant effects (p < 0.05) are indicated in boldface. L, Left; R, Right; B, Bilateral; ME, Main Effect; INTX, Interaction.*

The reduced activation with repeated exposures to stimuli may have represented participant failure to respond to stimuli or overall fatigue. As the behavioral orienting task was a subjective judgment, we were not able to calculate an accuracy rate to determine if accuracy decreased with the duration of the task. Nevertheless, response rates remained high (>94%) throughout the course of the task. As a check for overall fatigue, we modeled the single-presentation general computing screenshots. If the observed decreases in activation were due to overall fatigue, the effect should generalize to the novel stimuli as well. In all clusters of activation the activity for the novel stimuli was greater than for the final presentation of either the general computing or warning stimuli (Figure 3), with the sole exception of the warning stimuli in the right anterior insula.

**FIGURE 4:**
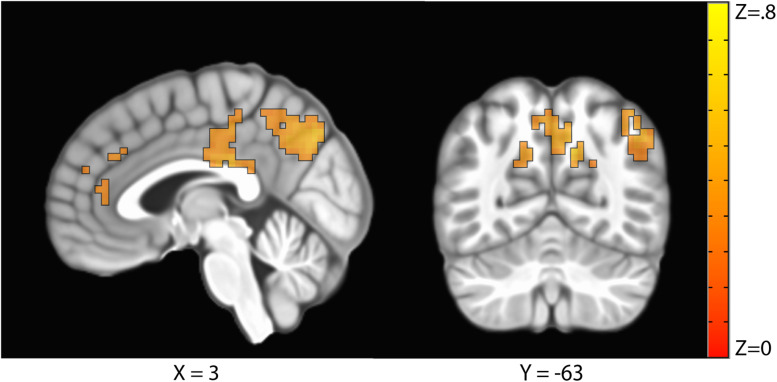
Clusters where activation was significantly correlated with the precuneus in a representational similarity analysis (RSA) included the precuneus, the posterior cingulate cortex, the right temporal parietal junction, the medial prefrontal cortex, and the right frontopolar cortex.

The sustained negative linear trends in the majority of clusters of activation are consistent with habituation processes. Conversely, activation in the right precuneus increased with repeated exposures to the stimuli. The precuneus is a hub of the default mode network (DMN; Raichle, 2015), which is a network of brain structures that become more active as participants engage less in a primary task (Mason et al., 2007). To test whether the increasing activation observed in the precuneus represented DMN activation, we conducted a similarity analysis by extracting the mean betas for each condition in the precuneus cluster and calculating a correlation with this pattern of activation across every voxel in the brain. Correlation coefficients were Fisher transformed and a *t*-test was performed on these values versus 0 to identify regions where activation was significantly correlated with that of the precuneus. Five clusters were identified: the precuneus, posterior cingulate cortex, right temporal parietal junction, medial prefrontal cortex, and right frontopolar cortex (Figure 4 and Table 3). As these regions are commonly associated with the DMN (Raichle, 2015), we conclude that the increasing activation with stimulus repetition observed in the precuneus likely reflects increased DMN activation.

**TABLE 3:** Clusters significantly correlated with activation in the precuneus.

	**Voxels**	**MNI coordinates**		
**Label**		***X***	***Y***	***Z***
B. Precuneus	265	9	−70	37
R. Temporal parietal junction	118	50	−52	40
B. Posterior cingulate cortex	76	2	−28	27
R. Frontopolar cortex	46	22	58	−8
B. Medial prefrontal cortex	42	2	40	13

*L, Left; R, Right; B, Bilateral.*

## Discussion

In the current experiment, participants viewed repeated images of software applications and security warnings while they underwent fMRI. We found evidence of repetition suppression for both stimulus types throughout the visual processing stream. Critically, fMRI activation continued to decrease over all six repetitions of the stimuli, indicating a continued repetition suppression effect with continued stimulus exposures. Conversely, we observed increased activation in DMN regions with repeated exposures. Finally, we observed increased activation in frontal regions including the pre-SMA, inferior frontal gyrus, and anterior insula for security warning stimuli compared to general software applications, consistent with heightened negative subjective value for the warning stimuli. These findings indicate that repetition suppression is multifaceted, differentially affecting a variety of areas.

We first examined the repetition suppression effect to everyday stimuli. We observed distinct patterns of activation over the course of repetitions. Similar to previous studies, there was a decrease of activation in areas related to visual processing, namely in the occipital lobe (Kovacs et al., 2013; Mayrhauser et al., 2014) and inferior temporal lobe (Summerfield et al., 2008). Adding to this previous work, we observed a continued, linear decrease in activation through all six repetitions. Such a finding shows that the decrease of activation in these areas does not level off after the second trial but continues to decrease with prolonged exposure to the stimulus. This repetition suppression occurred in frontal regions as well and applied to both images of general computing software and security warnings, indicating that the learned negative valence of computer security warnings is not enough to overcome habituation.

Along with the decreased activation in the occipital and inferior temporal lobes with repeated presentations, we also observed increased activation in the DMN, namely the precuneus and PCC. Activation in the DMN has been demonstrated to be negatively correlated with activation in a network of regions known to be involved in directing external attention, the dorsal attention network (Fox et al., 2005). Thus, increased activation in the DMN is often associated with unconstrained mental activity (“mind wandering”) (Mason et al., 2007; Raichle and Snyder, 2007). The continued increased activation in the DMN during subsequent stimulus presentations suggests that the participants were less attentive to the stimuli as the repetitions increased.

Because we used naturalistic stimuli with learned negative valence, we were able to examine the differential response to positive and negative valance images over several repetitions. We observed greater activation for general software stimuli than the warning stimuli in posterior regions including the bilateral visual stream and retrosplenial cortex. The general software images were on average larger (general software mean image dimensions: 760.8 × 1,173.4 pixels; warning mean image dimensions: 381.9 × 589.4 pixels), which might have accounted for some of the greater activation in the visual processing stream. To control for this, we entered stimulus size on each trial as a nuisance regressor in the first-level regression analysis. In spite of this control, we nevertheless observed widespread activation differences between stimulus types in the visual processing streams. This could be explained by elements of the images as the general software stimuli contained images used for work and recreation providing various uses, options, and tools. In contrast, the security warnings were less captivating with a lack of information and visual stimuli within the image. In spite of this, the linear trend interaction between stimulus types in the visual processing streams and intraparietal sulcus indicates that any additional visual or attentional processing afforded the general computing images habituated faster (i.e., had a steeper negative linear trend) than security warnings.

For areas including inferior frontal gyrus, pre-SMA, and right anterior insula, there was a greater level of activation for the computer warning stimuli than the general software images. The anterior insula has been specifically associated with anxiety and fear conditioning (Grupe and Nitschke, 2013) and has been implicated in initiating a fear response as a result from negative or harmful stimuli (Knutson et al., 2014). The greater activation for computer warning stimuli as opposed to general software images in this region is consistent with a fear response to the warning stimuli over the general software stimuli. An anterior insula-mediated fear response functions not only for environmental risk, but also for safety from other negative experiences and stimuli (Knutson et al., 2014). Additionally, the anterior insula response was still activated even though the computer warning stimuli was fictitious. The participant was informed before participation that the computer warnings were mock images and not directly related to them or their property. This is consistent with other studies that have shown that fear response is still activated even when not part of the primary task (Carlsson et al., 2004).

Some limitations should be noted in the present study. First, while we examined the repetition suppression effect with complex stimuli, we looked at these stimuli with repeated repetitions within a short period of time. The use of complex stimuli adds to the external validity of the study, but computer security warnings are generally observed infrequently over longer periods of time (days or weeks). A longitudinal study looking at how extended exposure over several weeks could add to the findings of this study by presenting these stimuli in a more natural time course. Second, computer security warnings are a familiar sight among individuals who regularly use computers. Further, we did not assess the pre-experimental familiarity of the stimuli in this group of participants. Therefore, these stimuli may not have been completely novel. Regardless, we still found a strong repetition suppression effect even when the participants had encountered similar stimuli previously in everyday use of computers. This suggests a potential line of research examining the extent to which habituation generalizes from non-security messages to computer security warnings. In other words, future studies may wish to examine whether participants habituate to innocuous system notifications (such as email notifications) and whether that habituation generalizes to security warnings. Third, we do not determine the number of repetitions where activation begins to level off. While other research shows that the greatest decrease in activation occurs during early repeated exposures to stimuli to complex (Anderson et al., 2016a,b; Vance et al., 2018), future research is needed to determine at what point additional repetitions do not cause a meaningful decrease in activation. Finally, we did not collect valance ratings or physiological arousal measurements associated with the warning stimuli. However, previous studies (e.g., Buck et al., 2017) have demonstrated negative valance associated with pop-up security warnings.

One strength of this study is that it examined the repetition suppression effect in complex, everyday stimuli as well as examining this phenomenon with extended repetitions. This design allowed us to replicate and confirm previous findings of earlier research that visual processing activation decreases over repetition as well as DMN activation increases over repetition. Along with confirming prior research on the subject, the use of complex stimuli allows these findings to be generalized to greater variations of stimuli than have been used in prior research. Finally, we demonstrated that the anterior insula responded to the negative valence of the computer warning stimuli and that this increased activation also demonstrated a repetition suppression effect over continued exposures. The habituation to warnings is a concern for computer security as users are less likely to attend and respond appropriately to repeated computer security warnings.

## Data Availability Statement

The datasets generated for this study are available at https://doi.org/10.18112/openneuro.ds002363.v1.1.1. The code used in the analyses presented is available at https://github.com/Kirwanlab/RepetitionSuppression.

## Ethics Statement

The studies involving human participants were reviewed and approved by Brigham Young University Institutional Review Board. The patients/participants provided their written informed consent to participate in this study.

## Author Contributions

CBK, BA, AV, DE, and JJ conceptualized the experiment. DB, BA, AV, DE, and JJ collected stimuli and designed the experimental paradigm. CBK, DB, BA, and AV collected the data. CBK and DB performed the data analysis. CBK, DB, BA, AV, DE, and JJ edited and wrote the manuscript. All authors contributed to the article and approved the submitted version.

## Conflict of Interest

The authors declare that this study received funding from Google. The funder was not involved in the study design, collection, analysis, interpretation of data, the writing of this article or the decision to submit it for publication.

## Footnotes

1 https://github.com/rordenlab/dcm2niix
